# Prostate Cancer, JAK/STAT3 Dysregulation, and Flavonoids: Is There a Possible Link?

**DOI:** 10.3390/ijms27020885

**Published:** 2026-01-15

**Authors:** Valentina Uivarosi, Daniela Miricescu, Ileana Adela Vacaroiu, Dan Arsenie Spinu, Constantin Stefani, Silviu Stanciu, Remus Iulian Nica, Iulia-Ioana Stanescu-Spinu, Silviu Constantin Badoiu, Silvia Nica, Viorel Jinga

**Affiliations:** 1 Department of General and Inorganic Chemistry, Faculty of Pharmacy, Carol Davila University of Medicine and Pharmacy, 6 Traian Vuia Str., 020956 Bucharest, Romania; valentina.uivarosi@umfcd.ro; 2 Discipline of Biochemistry, Faculty of Dentistry, Carol Davila University of Medicine and Pharmacy, 8 Eroii Sanitari Blvd., 050474 Bucharest, Romania; 3 Department of Nephrology, Faculty of Medicine, Carol Davila University of Medicine and Pharmacy, 8 Eroii Sanitari Blvd., 050474 Bucharest, Romania; 4 Department 3, Faculty of Medicine, Carol Davila University of Medicine and Pharmacy, 8 Eroii Sanitari Blvd., 050474 Bucharest, Romania; arsenie.spinu@umfcd.ro; 5 Urology Department, “Dr. Carol Davila” Central Military Emergency University Hospital, 134 Calea Plevnei, 010825 Bucharest, Romania; 6 Department I of Family Medicine and Clinical Base, “Dr. Carol Davila” Central Military Emergency University Hospital, 134 Calea Plevnei, 010825 Bucharest, Romania; constantin.stefani@umfcd.ro; 7 Department of Internal Medicine and Gastroenterology, Carol Davila University of Medicine and Pharmacy, “Dr. Carol Davila” Central Military Emergency University Hospital, 134 Calea Plevnei, 010825 Bucharest, Romania; silviu.stanciu@umfcd.ro; 8 Discipline of General Surgery, Faculty of Midwifery and Nursing, Carol Davila University of Medicine and Pharmacy, 8 Eroii Sanitari Blvd., 050474 Bucharest, Romania; remus.nica@umfcd.ro; 9 Surgery Department, “Dr. Carol Davila” Central Military Emergency University Hospital, 134 Calea Plevnei, 010825 Bucharest, Romania; 10 Discipline of Physiology, Faculty of Dentistry, Carol Davila University of Medicine and Pharmacy, 8 Eroii Sanitari Blvd., 050474 Bucharest, Romania; iulia.stanescu@umfcd.ro; 11 Department of Anatomy and Embryology, Faculty of Midwifery and Nursing, Carol Davila University of Medicine and Pharmacy, 8 Eroii Sanitari Blvd., 050474 Bucharest, Romania; silviu.badoiu@umfcd.ro; 12 Department of Emergency and First Aid, Carol Davila University of Medicine and Pharmacy, 8 Eroii Sanitarii Blvd., 050474 Bucharest, Romania; silvia.nica@umfcd.ro; 13 Emergency Discipline, University Hospital of Bucharest, Independence Str., 050094 Bucharest, Romania; 14 Department of Urology, Carol Davila University of Medicine and Pharmacy, 8 Eroii Sanitari Blvd., 050474 Bucharest, Romania; viorel.jinga@umfcd.ro; 15 Romanian Academy, 125 Calea Victoriei, 010071 Bucharest, Romania

**Keywords:** prostate cancer, JAK/STAT3 dysregulation, flavonoids

## Abstract

Worldwide, prostate cancer (PC) has a rising incidence and is the sixth leading cause of death globally, especially with increasing cases in developing countries. Risk factors for PC include genetic predisposition, family history, race/ethnicity, and various occupational factors like diet, obesity, smoking, and transmitted diseases. The Janus kinase (JAK)-signal transducer and activator of transcription (STAT) pathway can be activated by hormones, cytokines, and growth factors, and it plays a role in many vital biological processes such as cell growth, differentiation, immune regulation, and apoptosis. Dysregulation of JAK/STAT3 can lead to cancer, inflammation, diabetes, and neurodegenerative disorders. In cancers, including PC, STAT3 promotes cell survival, progression, angiogenesis, and metastasis. Inhibitors targeting JAK and STAT3 tested in vivo have shown potential to inhibit malignant cell growth. Additionally, flavonoids are bioactive plant compounds that are important in preventing inflammation, oxidative stress, and cancer. Research indicates that natural flavonoids can be developed into cancer-preventive and therapeutic agents. Experimental studies have demonstrated that some flavonoids can inhibit PC development. The main goal of this review is to present the incidence and risk factors of PC, the JAK/STAT3 pathway and its inhibitors, and how flavonoids may influence this pathology.

## 1. JAK/STAT3 Pathway Overview

In cells, transcription factors are vital because they regulate the expression of genes that encode proteins involved in cell communication, metabolism, immune responses, and the cell cycle [1]. The Janus kinase (JAK)-signal transducer and activator of transcription (STAT) pathway has three components—cell-surface receptors, JAK proteins, and STAT proteins [2]—all of which play roles in various biological processes such as cell growth, differentiation, immune regulation, and apoptosis [3]. JAKs belong to a family of non-receptor tyrosine kinases, and STATs are inactive cytosolic transcription factors that transmit signals from the cell membrane to the nucleus [4,5].

The JAK family includes four receptor-associated kinases, JAK1, JAK2, JAK3, and TYK2 (tyrosine kinase 2), while the STAT family is composed of seven proteins: STAT1, STAT2, STAT3, STAT4, STAT5A, STAT5B, and STAT6 [6].

JAK1, JAK2, and TYK2 are expressed throughout the body, while JAK3 is primarily found in myeloid and lymphoid cells, which are part of the hematopoietic system [7]. The JAK family members have four domains, including the SH2 domain, which links the receptor to the STAT protein [8]. The four structural domains of JAKs include regions called JH1 through JH7. JH1 and JH2 are the kinase and pseudokinase domains. JH2 plays a regulatory role rather than a catalytic one. JH2 inhibits ligand-independent kinase activity through direct interactions with JH1 but is also essential for ligand-induced JAK activation. JH3 and JH4 primarily support the enzyme’s structural stability. Meanwhile, the JH5, JH6, and JH7/FERM domains—also known as Four-point-one protein, Ezrin, Radixin, and Moesin—are crucial for JAKs’ association with their respective receptors [9]. In the cytoplasm, some receptors for tyrosine residues are phosphorylated by activated JAK, creating docking sites for the subsequent binding of STAT components [8].

Hormones, chemokines, and growth factors activate the JAK/STAT3 signaling pathway [9]. When a cytokine or growth factor binds to its receptor, it activates the associated tyrosine kinases known as JAK1–3 and TYK2, which then trigger the activation of the STATs [10]. STAT3 activation can be triggered by various growth factor receptors including hepatocyte growth factor receptor (HGFR), epidermal growth factor receptor (EGFR), and platelet-derived growth factor receptor (PDGFR), as well as by non-receptor cytoplasmic tyrosine kinases like Abelson leukemia protein and Src-related kinases [11]. Moreover, STAT3 activation occurs when cytokines or peptide hormones bind to cell-surface receptors, initiating a series of events that include phosphorylation at key sites, mainly tyrosine (Y) at position 705. This process results in the activation or reorganization of the dimer from NTD-to-NTD to SH2-to-SH2, nuclear localization, and transcriptional activation of specific genes through binding to their promoter DNA elements [12]. First, cytokines bind to their specific transmembrane receptors, causing receptor dimerization and JAKs’ activation, which then associate with and phosphorylate the receptors. Next, tyrosine residues in the receptor’s catalytic domain are phosphorylated, creating a docking site where STAT proteins with SH2 domains are recruited. The STAT proteins are subsequently phosphorylated and form either homodimers or heterodimers. In the dimerized form, STATs dissociate from their receptors and move into the nucleus, where they bind to DNA sites and control gene transcription [2]. Therefore, STAT activation is highly complex owing to the regulatory epigenetic mechanisms that govern various cellular functions [13].

Somatic mutations in JAK/STAT pathway genes are recognized as cancer drivers. Activating mutations in JAK are mostly found in hematological cancers but also occur in some non-hematological cancers. STAT3 is the most frequently mutated STAT in cancer. Similarly to JAK2, STAT3 mutations are common in hematological tumors, but are also present in various solid tumors [10]. JAK/STAT dysregulation can lead to cancer, inflammation, neurodegenerative disorders [11], obesity, diabetes, and other metabolic diseases [12].

## 2. Prostate Cancer Epidemiology: Incidence, Risk Factors, and Development

Worldwide, prostate cancer (PC) is the second most common cancer and the sixth leading cause of cancer-related deaths. By 2040, the global incidence of PC is expected to reach nearly 2.3 million new cases and 740,000 deaths, mainly due to population growth and aging [13]. Its incidence is increasing in many countries, especially in developing ones, whereas mortality is decreasing in developed nations [14].

In Northern Europe, North America, and Australia, incidence rates are the highest, mainly due to the widespread use of PSA-based testing for early detection. Conversely, the highest mortality rates are seen in Sub-Saharan Africa, Latin America, and the Caribbean, where individuals of African ancestry—who are genetically more susceptible to PC—have limited access to diagnosis and treatment [15]. In Canada, PC is the most common cancer and the third leading cause of death among men [16]. When comparing the incidence and mortality rates of PC between men in rural and urban areas, most reports indicate higher rates in urban men because rural men are less likely to undergo screening and, as a result, are less often diagnosed. Consequently, rural men experience higher death rates compared to their urban counterparts [17]. Additionally, a higher incidence has been observed among black men of the African diaspora in the United States and the Caribbean [18].

A combination of genetic and environmental factors explains the ethnic and geographical differences in PC incidence and mortality rates [19]. The main risk factors for PC include age, ethnicity, and genetics. Therefore, variations in the expression of the *ELAC2*, *RNASEL*, *MSR1*, *BRACA2*, and *HOXB13* genes, along with a low number of CAG repeats in the androgen receptor gene, are involved in PC development [20,21,22,23,24,25]. Studies show that up to 80% of cases are diagnosed in men over the age of 65 [16].

The risk of a PC-positive family history is about 20%, with at least one first-degree relative having PC [26]. Familial PC (FPC) is defined as having either two first-degree relatives diagnosed with PC at any age, or one first-degree relative and two or more second-degree relatives diagnosed at any age [27]. FPC has the highest heritability among all primary cancers in men [25], and it helps identify families with a powerful history of PC [27]. This includes those with three or more affected first-degree relatives, cases where PC is diagnosed in three consecutive generations of the same lineage (paternal or maternal), or two first-degree relatives both diagnosed with early-onset disease (≤55 years) [27].

Besides genetic predisposition, various risk factors, such as family history and race/ethnicity, as well as a range of individual, environmental, and occupational factors, have been suggested to explain differences in the epidemiological burden of this disease [28,29,30]. Prostatic malignant tissue is often linked to dysregulation of essential metals such as zinc, copper, and iron. Additionally, epidemiological studies have shown that this dysregulation, along with excessive exposure to certain non-essential heavy metals [31,32] or air pollutants such as benzene, SO_2_, NO, CO, NO_2_, toluene, and O_3_, may contribute to PC development [33].

Furthermore, sexually transmitted diseases, obesity, smoking, alcohol use, vasectomy, and diet are other risk factors for PC [34]. Early sexual activity and sexually transmitted infections—both viral (HSV-2, HPV-18, and HPV-16, CMV) and bacterial (*Neisseria gonorrhoeae*, *Treponema pallidum*, *Chlamydia trachomatis*)—may significantly contribute to PC development [20].

Consuming more fats, proteins (read meat), dairy products, and carbs can have negative effects because higher intake is linked to inflammation [33,35,36,37,38,39]. The periprostatic adipose tissue near the prostate is recognized as a key factor in disease progression [40]. It is well known that excess fat mass causes low-grade chronic inflammation, which leads to abnormal secretion of adipokines and disrupts immune responses and other metabolic processes [41]. Growing evidence indicates that adipocytes supply lipids used by nearby PC cells [42]. Both elevated circulating leptin levels and leptin receptor mutations are associated with a higher risk of PC in humans [43]. Therefore, the consequences of obesity, including hormonal imbalance, persistent inflammation, and oxidative stress, can be linked with PC development [44].

The connection between PC, smoking, and alcohol consumption shows inconsistent results [45,46]. Former smokers face a higher risk of PC development due to cadmium released from cigarette smoke [45]. Depending on the type of alcohol consumed, it can promote prostate tumor growth and significantly reduce the time to progression to metastatic PC [47].

Research indicates that changes in microbiota composition can significantly influence the development, progression, and prognosis of PC, because various microbes lead to genotoxin secretion causing mutagenesis or promoting tumorigenic inflammation and impaired immunosurveillance [48,49,50].

The prostate gland relies on androgens for growth and development [51]. Leydig cells from the testes produce testosterone, which is regulated by luteinizing hormone (LH) released from the anterior pituitary gland [52]. Testosterone circulates bound to serum sex hormone-binding globulin (SHBG) and albumin. Only the free form of testosterone can enter prostate cells, where it is converted into 5α-dihydrotestosterone (DHT), essential for prostate cell growth and survival [52]. Under healthy conditions, the androgen receptor (AR) promotes communication between epithelial and stromal cells, maintains structural integrity, and preserves the prostate’s immune-privileged status against inflammation and autoimmunity, which is essential for prostate gland function [53].

AR deregulation or androgens lead to PC [54]. Therefore, the progression of PC depends on androgen hormones, which bind to AR to activate tumor proliferation pathways [55]. After binding, the receptor dissociates from accessory proteins, translocates into the nucleus, dimerizes, and then binds to the androgen response element (ARE) located in the promoter regions of genes involved in cellular proliferation and evading apoptosis [56]. Since the androgen axis is essential for tumor growth, treatment strategies often involve blocking this signaling pathway through surgical or medical castration [57].

For PC to develop, prostate cells become cancerous through multiple stages, starting with PIN (prostatic intraepithelial neoplasia), progressing to localized PC, and eventually advancing to prostate adenocarcinoma and metastasis [58].

PC and benign prostatic hyperplasia (BPH) are among the most common urological conditions affecting older men [59]. PC shares several features with BPH and the presumed precursor to cancer, prostatic intraepithelial neoplasia (PIN). All these conditions become more common with age, all depend on androgens for growth and development, and all respond to androgen-deprivation therapy [60].

Although most PC cases begin as androgen-dependent hyperplasia, mutations and selective pressure gradually reduce their reliance on AR stimulation, leading to the development of androgen-independent PC, which is much more aggressive and associated with poor outcomes [61]. Prostate adenocarcinoma can progress to metastatic (or advanced) PC, a severe malignancy that may spread to the lymph nodes, lungs, or other organs [61]. Neuroendocrine prostate cancer (NEPC) is a deadly subtype of PC. NEPC very rarely arises de novo; instead, it mainly develops from adenocarcinoma as a response to drug-induced androgen receptor signaling inhibition [62]. Metastatic castration-resistant prostate cancer (mCRPC), marked by resistance to AR-targeted therapies, develops quickly, and further disease progression is often unavoidable. mCRPC is characterized by various genomic and transcriptional abnormalities, including EGFR-mutant lung adenocarcinoma, BRAF-mutant melanoma, and estrogen receptor-positive breast cancers [63].

PC can be either slow-growing or aggressive. While slow-growing tumors may remain hidden and cause no symptoms for a lifetime, aggressive tumors can quickly progress to more severe stages [64]. Fatal PC is defined as death from the disease within 10 years of diagnosis [65].

## 3. JAK/STAT3 Dysregulation and Prostate Cancer: From Molecular Mechanisms to Therapeutics

### 3.1. Molecular Mechanisms

Chronic inflammation caused by infections, inflammatory diseases, altered metabolism, or other environmental factors significantly contributes to the development of various types of cancer, including PC [66]. The microenvironment linked to obesity increases growth factors and pro-inflammatory cytokines, which help promote invasion, metastasis, and androgen-independent growth [67]. Inflammation plays a crucial role in several stages of the metastatic process, supporting their survival, migration, invasion, and growth [68].

Chronic inflammation is frequently observed in prostate tumors. This inflammatory response involves the recruitment of leukocytes, such as myeloid cells, macrophages, and lymphocytes, to the prostate [69]. Immune cells infiltrating the prostate include those involved in innate immune responses, such as macrophages, neutrophils, mast cells (MCs), and natural killer (NK) cells, as well as cells associated with adaptive immune responses, like T- and B-lymphocytes [70]. Chronic inflammation is present in both benign and malignant prostate tissues [70]. Macrophages are among the most common components of the tumor microenvironment (TME), constituting 30–50% of all infiltrating inflammatory cells. Macrophages play a vital role in the TME [71]. They can polarize into either the classically activated M1-type or the alternatively activated M2-type, depending on various cytokines and growth factors, including IL-4, IL-13, and interferon-γ (IFN-γ) [71]. M2 macrophages help reduce inflammation and adaptive Th2 immunity while promoting angiogenesis. M2-type tumor-associated macrophages (TAMs) frequently infiltrate tumors and support malignant progression [72]. The M2 subtype is the dominant phenotype among tumor-promoting TAMs (pTAMs), creating an immunosuppressive TME that facilitates malignant growth and metastasis [71]. Furthermore, it has been suggested that TAMs release immunosuppressive cytokines [40]. Chemokines either promote or inhibit cancer cell growth, prevent apoptosis, and are crucial for cancer cell migration, which is necessary for metastasis. They also guide the movement of immune cells that act as chaperones at inflammation sites, and once activated, they initiate an immune response [73].

Within TME, IL-6 is produced by various cell types, including tumor-infiltrating immune cells, stromal cells, and the tumor cells themselves. IL-6 activates tumor cells to induce the expression of STAT3 target genes, which in turn encode proteins that promote tumor growth (such as cyclin D1) and/or survival (such as BCL-2-like protein 1 (BCL-xL) [74]. IL-6 has been detected in benign prostatic tissue, especially in basal cells. In PIN and cancer tissue, atypical intraluminal cells and cancer cells can express IL-6. Moreover, the expression of the IL-6 receptor (IL-6R) was detected in both benign and malignant tissues within the epithelium and stroma [75].

IL-6 plays a role in regulating immune responses as well as in cell growth and differentiation. Following the canonical pathway, IL-6 binds to its alpha IL-6R on the cell surface, leading to triggering homodimerization and association with the signal-transducing beta receptor glycoprotein 130 (gp130, encoded by Il6st) [76]. The activated IL-6R-gp130 complex engages and activates the JAK family—mainly JAK1, JAK2, and TYK2—which then phosphorylates the cytoplasmic tail of gp130, leading to the recruitment of STAT proteins, primarily STAT1 and STAT3 [76,77]. Deichaite et al. identified local IL-6 production in androgen-independent PC cell lines, indicating its role in autocrine and paracrine functions [78].

In PC, the JAK/STAT signaling pathway can be activated by various cytokines, besides IL-6 and growth factors [79], and may regulate hormone secretion, growth, and inflammation. Active STAT3 promotes cell survival by increasing the transcription of anti-apoptotic genes (Bcl-xL and Survivin) and genes involved in cell cycle progression (c-myc and Cyclin D1), angiogenesis [(vascular endothelial growth factors) VEGF and HIF-1α)], and immune evasion (RANTES) [80].

STAT-3 hyperactivation and its movement into the nucleus promote increased cell growth, progression through the cell cycle, and resistance to cell death. IL-6 triggers inflammation and also regulates MAPK and JAK/STAT cancer-related pathways [81]. STAT3 is a key downstream regulator of IL-6, playing an essential role in adaptive growth and cancer development in PC [81]. Activation of various signaling pathways, such as JAK/STAT 3, mitogen-activated protein kinase (MAPK), and phosphatidylinositol 3-kinase (PI3K), by IL-6 has been reported in multiple PC cell lines. It has been shown that IL-6 activates the AR in PC, even in the absence of androgen [40]. IL-6 regulates the expression of VEGF and the neuroendocrine differentiation process. It appears that IL-6 is implicated in the epithelial/mesenchymal transition (EMT)/metastasis of PC [40].

**Figure 1:**
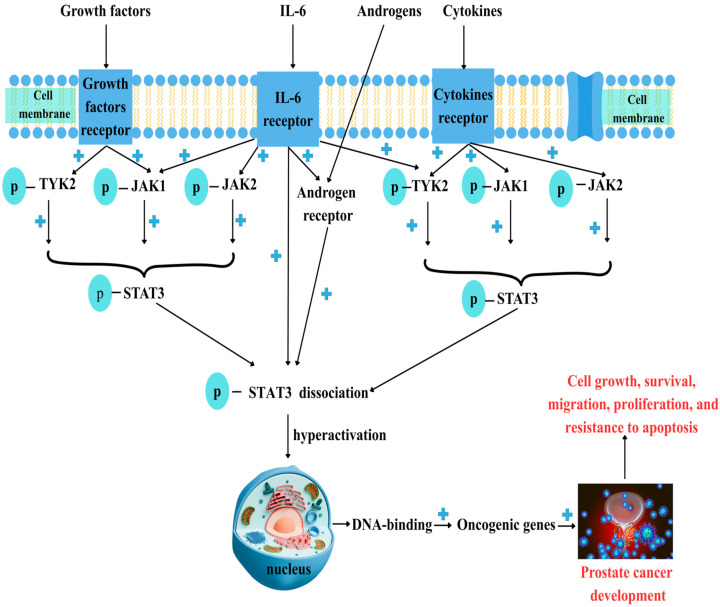
Prostate cancer and JAK/STAT3 dysregulation. Prostate cancer (PC) development involves various factors such as growth factors, cytokines like IL-6, and androgens. After binding to their specific receptors, Janus kinase receptors (JAK1, JAK2, and TYK2) are phosphorylated, which then activates signal transducer and activator of transcription 3 (STAT3). Androgens bind to their specific receptor (AR) and promote STAT3 phosphorylation (p-STAT3). Phosphorylated, active STAT3 dissociates from JAKs and moves into the nucleus, where it binds to DNA. STAT3 hyperactivation is linked to malignant transformation, leading to the activation of oncogenic genes upon DNA binding and promoting cell growth, survival, migration, differentiation, and resistance to apoptosis. “+”—activation.

These molecular events are in concordance with serum modifications, where levels of IL-6 are increased in patients with hormone-refractory PC [82,83]. Serum levels of IL-6 are higher in patients with untreated metastatic or castration-resistant prostate cancer (CRPC) and are inversely related to tumor survival and chemotherapy response [84]. Furthermore, IL-6 is associated with an aggressive PC phenotype and may facilitate metastasis by regulating EMT and the invasion of cancer cells into the bone [84]. In the tumor microenvironment across various cancer types, IL-6/JAK/STAT3 signaling promotes tumor cell growth, survival, invasiveness, and metastasis, while significantly suppressing the antitumor immune response [74] (Figure 1). Therefore, the proinflammatory cytokine IL-6 is linked to poor prognosis in PC and plays a role in progression to castration resistance [85]. Studies involving men with mCRPC reported that tumors from African American patients showed decreased levels of IFN-γ, IL-6/JAK/STAT3, and inflammatory pathway genes compared to tumors from European American patients [86]. Data from 202 PC patients showed that levels of pJAK-1 (Tyr1022/1023) and pSTAT-3 (Tyr705) were both positively associated with Gleason score and clinical stage. Their expression was also significantly higher in patients with increased biochemical PSA levels [14].

Furthermore, in vivo and in vitro studies show that the NF-κB-IL6-STAT3 axis activated by intratumoral lipopolysaccharide (LPS) drives PC growth [87]. It is well known that the NF-κB pathway is a key regulator of inflammation and immune responses, and its dysregulation contributes to malignant development and progression [87,88].

STAT3 controls the expression of various genes in response to cellular signals, making it essential for cell growth and apoptosis. The activation of and interaction between STAT3 and NF-κB are vital for regulating communication between cancer cells and inflammatory cells. NF-κB and STAT3 are two key factors that help pre-neoplastic and malignant cells resist apoptosis-driven tumor surveillance and manage tumor angiogenesis and invasiveness [89].

STAT3 is activated by the entire family of IL-6-type cytokines, including IL-6, IL-11, IL-22, IL-27, IL-31, cardiotrophin 1, oncostatin M, ciliary neurotrophic factor, leukemia inhibitory factor, and cardiotrophin-like cytokine factor 1. These cytokines are involved in embryonic development, immune response, inflammation, blood cell formation, cardiovascular health, liver function, and nerve repair [90].

Interleukin-17 (IL-17) is a key proinflammatory cytokine primarily secreted by immune cells. Currently, six members of the IL-17 family have been identified, ranging from IL-17A to IL-17F [91]. Furthermore, the IL-17 receptor (IL-17R) family consists of five members: IL-17RA, IL-17RB, IL-17RC, IL-17RD, and IL-17RE. Studies have shown higher levels of IL-17A and IL-17RA expression in PC cell lines, which promote growth and metastasis even during castration conditions [91].

Dysregulation of the JAK/STAT pathway in PC can lead to increased programmed death-ligand 1 (PD-L1) expression [92]. It is well known that the PD-1/PD-L1 signaling pathway plays a role in controlling tumor immune escape by affecting various immune cells, including T- and B-lymphocytes, NK cells, macrophages, dendritic cells, and other components of the TME [92]. PD-1/PD-L1 suppresses the function and activation of tumor-infiltrating lymphocytes (TILs) and NK cells, promotes TILs apoptosis, and impacts the differentiation of T helper and myeloid cells [92]. Kazan O and colleagues, performing radical prostatectomy, revealed that among 100 patients, 9 of 11 patients with PDL-1 expression also showed intermediate to high pSTAT-1 staining intensity, and those with PDL-1 expression displayed higher pSTAT-1 staining intensity than those without [93].

JAK-STAT pathway activation is regulated by several molecules, especially the suppressor of cytokine signaling (SOCS) family, which includes eight members: SOCS1 through SOCS7 and the cytokine-inducible SH2-containing (CIS) proteins [94]. Suppressor of cytokine signaling 3 (SOCS3) acts as an IL6-induced negative feedback regulator of the IL6/JAK/STAT3 pathway. The SOCS3 promoter is hypermethylated in cancerous regions compared to adjacent benign tissue in PC [95]. SOCS3 dysregulation enhances JAK/STAT activity due to abnormal cytokine signaling, leading to increased cancer cell proliferation, differentiation, and migration [85]. Adenoviral vectors delivering the SOCS3 gene have been shown to boost the sensitivity of PC with JAK/STAT3 overactivation to NK cells by reducing PD-L1 levels and IL-6 production [92].

Long noncoding RNAs (lncRNAs) are types of endogenous cellular RNA longer than 200 nucleotides that do not encode proteins. Research has shown that abnormal expression of lncRNAs can either promote or inhibit tumor development in various types of cancer [96]. Studies have shown that several lncRNAs, including LOXL1-AS1, PVT1, and HOTAIR, exhibit abnormal expressions in PC. These lncRNAs have complex roles in tumor development, such as promoting cell proliferation, migration, and apoptosis. Xing Z and coworkers demonstrated that LINC00473 is involved in regulating cell growth and expression in the JAK-STAT3 signaling pathway [96].

Unfortunately, JAK/STAT signaling is crucial for the long-term survival and growth of various cancer cells and may also be linked to cancer cell resistance to different chemotherapies [97]. Therefore, activating STAT3 signaling is vital for the metastatic progression of PC [98]. Kroon P indicates that the most primitive cells in PC rely on pSTAT3 for survival, supporting STAT3 as a therapeutic target for treating advanced PC [99]. Additionally, targeting key components of IL-6 signaling, such as IL-6Rs, gp130, STAT3, and JAK, with monoclonal antibodies, remains a major challenge in preclinical cancer research [100].

### 3.2. JAK/STAT3 Inhibitors

Experimental studies involving in vivo and in vitro research focused on phosphorylated JAK/STAT3 inhibition, but also IL-6, because this cytokine induces their overexpression (Table 1). In this context, the anti-IL-6 antibody siltuximab (CNTO 328) has been demonstrated to decrease prostate tumor growth both in vitro and in vivo, and to slow progression toward castration resistance. Unfortunately, in phase II studies, the anti-IL-6 antibody was not an effective treatment for patients with metastatic PC [101]. Therefore, endogenous IL-6 inhibitors, such as cytokine signaling molecules and protein inhibitors of activated STAT, exert different effects on prostate cells depending on whether the AR is present or absent [102]. A study involving 20 patients scheduled for radical prostatectomy examined the effects of no drug versus siltuximab, which was administered once, twice, or three times before surgery [103]. The study confirms that patients did not report adverse events related to siltuximab. Furthermore, patients treated with siltuximab exhibited elevated levels of proliferation and apoptosis markers. After a single dose, serum concentrations of siltuximab decrease in a biexponential pattern [103]. The study also demonstrated a reduction in phosphorylation of STAT3 and p44/p42 mitogen-activated protein kinases. Additionally, gene expression analyses show downregulation of genes specific to the IL-6 signaling pathway and key enzymes in the androgen signaling pathway [103].

Bradley et al. conducted a phase II trial to evaluate the effectiveness of vorinostat in patients with metastatic castration-resistant PC who had previously undergone chemotherapy. The study involved 27 patients who received a specific oral dose of vorinostat daily. The results indicated that the dose of vorinostat caused significant toxicities, which limited the ability to fully assess its efficacy in this patient group. Vorinostat can disrupt the IL-6 signaling cascade [104].

Studies use cell lines and xenograft mouse models to test the inhibitory effect of Indirubin. Therefore, Indirubin can inhibit proliferation, induce apoptosis in endothelial cells, inhibit angiogenesis in vivo, and suppress VEGFR2-mediated JAK/STAT3 signaling. Handle et al. investigated how the small-molecule inhibitor galiellalactone blocks STAT3, leading to a significant decrease in AR activity in various PC cell lines. The study confirms galiellalactone’s effectiveness in primary tissue slice cultures from radical prostatectomy samples [95].

Shi C studied how borneol (BNL) affects the growth and death of human PC cells by inhibiting JAK and STAT-3 expression in human PC (PC-3) cells treated with various concentrations. The study found that BNL decreased IL-6, JAK1, and STAT3 phosphorylation in PC-3 cells, stopping the production of proteins involved in cell growth and preventing apoptosis [105].

Gurbuz et al. tested the effects of a JAK 2-AG490 inhibitor and a STAT3-S3I-201 inhibitor on PC cells treated with IL-6 in vitro. The study showed that IL-6 induced phosphorylation of STAT3. Furthermore, adding AG490 and S3I-201 to the IL-6-stimulated cells resulted in a time-dependent inhibition of STAT3’s tyrosine phosphorylation [106].

Studies conducted both in vitro and in vivo have demonstrated the potential therapeutic effects of garcinol in treating colon cancer, breast cancer, prostate cancer, head and neck cancer, and hepatocellular carcinoma. It mainly acts as an inhibitor of cellular processes by regulating transcription factors NF-κB and JAK/STAT3 in tumor cells. It has been shown to effectively inhibit the growth of malignant cell populations [107].

In vitro studies have demonstrated that tumor necrosis factor-related apoptosis-inducing ligand (Apo2L/TRAIL) or TRAIL antibodies, when combined with sorafenib, synergistically reduce cell growth and increase cell death in solid tumor cell lines, including breast, prostate, colon, liver, and thyroid cancers [108].

Enzalutamide is a second-generation anti-androgen that has demonstrated increased survival in patients with mPC. Hellsten et al. studied in vitro the effects of combining enzalutamide with the small-molecule STAT3 inhibitor GPB730 for enhanced therapeutic effects in advanced prostate cancer [109]. The PC cell lines LNCaP (androgen-dependent) and C4–2 (androgen-insensitive) were used. This study suggests that enzalutamide can be combined with the STAT3 inhibitor GPB730 to improve its efficacy, offering a new therapeutic approach to advanced PC [109].

The JAK1/2 inhibitor INCB018424 has shown significant clinical benefits, including reducing splenomegaly, night sweats, weakness, and irritation, which are linked to a decrease in proinflammatory cytokine release. Currently, INCB018424 is being investigated in the third phase of clinical trials [110].

Zerumbone exerts anticancer effects against hormone-refractory DU145 PC cells, possibly by inhibiting the abnormal IL-6/JAK2/STAT3 signaling pathway, and increases the sensitivity of DU145 cells to paclitaxel and other anticancer drugs [111].

**Table 1:** IL-6/JAKs/STAT3 inhibitors.

Inhibitor	Target	Study Type
*Siltuximab*	IL-6/p-STAT3	Phase II [101,102,103]
*Vorinostat*	IL-6	Phase II trial [104]
*Borneol*	JAK/STAT3	Cell lines [105]
*AG490*	JAK2	Cell lines [106]
*Enzalutamide + GPB730*	STAT3	Cell lines [109]
*Zerumbone*	IL-6/JAK2/STAT3	Cell lines [111]

Therefore, in the management of PC, besides PSA evaluation [112], imaging methods such as ^68^Ga-MY6349 and 18F-FDG PET/CT are used for various cancer types like PC [113,114]. Molecular characterization plays a crucial role.

## 4. Prostate Cancer, Flavonoids, and JAK/STAT Kinases: Is There a Connection?

Over the past several decades, plant-based products or phytochemicals have been recognized for their significant therapeutic potential [115]. Flavonoids are a group of compounds featuring two benzene rings, each with phenolic hydroxyl groups, connected by three central carbon atoms that form the C6-C3-C6 structure [116]. The primary dietary sources of flavonoids are fruits, vegetables, nuts, legumes, and plant-based beverages [117].

In nature, various types of flavonoids are present, originating from various plant sources and medicinal plants, including *Apium graveolens*, *Petroselinum crispum*, *Flemingia vestita*, and *Phyllanthus emblica* [118]. Flavones, flavanones, flavanols, flavonols, isoflavones, and anthocyanidins are the main subclasses of flavonoids, which are classified based on their structure. Most dietary flavonoids, except for flavan-3-ols, are found in the “glycoside” form [119,120].

In vitro and/or in vivo studies have demonstrated that flavonoids are vital nutrients for preventing various diseases such as cancer, inflammation, and bacterial infections [121]. They also help decrease the severity of neurodegenerative diseases, cardiovascular diseases, or diabetes [121]. Furthermore, flavonoids are known to regulate cellular metabolism, oxidative stress, cell cycle progression, angiogenesis, and epigenetic regulation, thereby preventing the development of diseases [122,123]. They are natural polyphenols with known anticancer and antioxidant effects. Growing evidence suggests that flavonoids help prevent cancer by lowering ROS levels [124,125]. Regarding ROS homeostasis, they play dual roles: they can function as antioxidants under normal conditions and act as strong pro-oxidants in cancer cells, triggering apoptotic pathways and reducing pro-inflammatory signaling pathways [126]. Natural flavonoids can act as lead compounds in developing cancer chemopreventive and/or therapeutic agents [127]. Additionally, they may disrupt the initiation, promotion, and progression of cancer [128]. Furthermore, research showed that the Japanese diet, which includes the intake of isoflavones and green tea, reduces the risk of localized and advanced PC [129]. Flavonoids can affect epigenetic changes, including those in oncogenes and tumor suppressor genes, which are indirectly controlled by epigenetic enzymes like DNA methyltransferase (DNMT), histone acetyltransferase (HAT), and histone deacetylase (HDAC) [130].

In 2016, around 58 flavonoids were reported to have anti-PC activity. In recent years, six additional flavonoid compounds have been identified as having the potential to combat PC [131]. Therefore, various chemopreventive flavonoids that may play a role in managing PC have been tested, including quercetin, luteolin, and apigenin [132]. Riale G conducted a population-based case–control study to examine the association between PC and dietary factors. The study involved patients with elevated PSA levels and/or suspected PC who were submitted for transperineal prostate biopsy (≥12 cores). The findings suggest that flavonols and catechins are the most promising compounds for potential protective effects against PC development [133].

Flavonoids can exert anti-androgenic effects through various mechanisms, such as inhibiting AR transactivators, decreasing AR activity, or directly competing with AR ligands. This is possible because of the structural similarity between these natural compounds and hormones [130].

### 4.1. Favonols

Flavonols, which have a distinctive hydroxyl group at the C3 carbon position, are the most common flavonoids found in foods. These compounds have been extensively studied in recent decades because they are convincingly linked to health benefits, including protection against diabetes and cardiovascular diseases [134]. Additionally, higher dietary intake of flavonols has been linked to a decreased risk of PC and bladder cancer [134].

Four main flavonols, including quercetin, myricetin, kaempferol, and fisetin, have been shown in laboratory studies to have chemopreventive effects in both castrate-resistant and castrate-sensitive PC models. The proposed mechanisms of flavonols’ action on the AR axis in PC include inhibition of 5α-reductase enzymes, direct androgen competition, suppression of AR complex formation, and transactivation by coregulators such as c-Jun, Sp1, and the PI3K/Akt pathway [135]. Quercetin glycoside is the most common flavonol, with properties that include anti-tumor, anti-ulcer, anti-allergy, antiviral, anti-inflammatory, and anti-diabetes effects. It also offers gastroprotection, lowers blood pressure, modulates the immune system, and fights against infections [136]. Quercetin has the potential to prevent the development of cancers in various solid tissues, including prostate, breast, kidney, lung, nasopharyngeal, colorectal, pancreatic, and ovarian cancers, as well as neurodegenerative diseases [136]. Quercetin inhibits STAT phosphorylation, thereby preventing their activation and nuclear translocation. Therefore, this inhibition interferes with the transcription of genes involved in cell growth and immune suppression, ultimately slowing down tumor progression [137].

Fisetin, a dietary flavonol, possesses cancer-preventive properties. A study tested the effects of fisetin on tumor necrosis factor-related apoptosis-inducing ligand (TRAIL), which may induce apoptosis in PC cells. The report states that fisetin increased the expression of TRAIL-R1 and decreased NF-κB activity [138]. Fisetin exhibits several pharmacological properties, such as antioxidant, anti-inflammatory, and anticancer functions. It has the capacity to inhibit JAK 1 and STAT3 signaling molecules in human thyroid cancer cells [139].

Kaempferol can inhibit the growth of both androgen-dependent and androgen-independent PC cells and induce apoptosis. Additionally, kaempferol causes cell cycle arrest at the G1 phase in 22Rv1 cells but at the S and G2 phases in PC-3 cells [140]. It was shown that kaempferol attenuates IL-6-induced COX-2 expression in human monocytic THP-1 cells, suggesting its beneficial role in chronic inflammation [141]. Astragalin or kaempferol 3-O-β-D-glucoside shows anti-inflammatory effects in vitro in macrophages, microglia, and epithelial cells by regulating complex signaling networks, including the JAK/STAT pathways [142]. Furthermore, myricetin and baicalin have been shown to promote DNA repair at a physiologically achievable concentration of 100 μmol/L [143]. Animal experiments conducted by Xu et al. tested the effects of icariin (ICA) on mice injected with tumor cells to inhibit PC development. Results showed that ICA, a natural flavonol, can inhibit PC neuroendocrine differentiation. The study reported that the combination of ICA and curcumin induces autophagy and ferroptosis in PC and affects lipid metabolism [144].

### 4.2. Flavanones

Eriocitrin, a lemon flavanone, exhibits antiproliferative and proapoptotic effects, and it can inhibit STAT3 phosphorylation by blocking an upstream molecule of JAK2 and Src kinase activation in breast cancer cells [145]. Naringenin, a natural flavanone antioxidant derived from citrus, influences PC cells (PC3 and LNCaP) by triggering apoptosis and increasing ROS production [146]. Additionally, naringenin may enhance the repair of oxidative DNA damage through the base excision repair pathway in PC cells [143]. Hesperidin, a flavanone glycoside, can inhibit PC cell growth by inducing apoptosis, regardless of whether cisplatin and paclitaxel are used. It also modulates the PI3K and MAPK signaling pathways in PC cells. Moreover, hesperidin boosts the release of Ca^2+^ from the endoplasmic reticulum (ER) [147]. Furthermore, Bakhshan et al. discovered that hesperidin nanoparticles not only demonstrated more potent prostate anticancer activity than free hesperidin, but also greater biocompatibility, with minimal toxicity to healthy cells [148]. Silibinin, a flavanone with anticancer and hepatoprotective properties, inhibits STAT3 phosphorylation and encourages apoptosis in prostate cancer cells [149]. Shao et al. studied the antitumor effects of the flavanone pinocembrin on human PC cells, focusing on apoptosis, endogenous ROS production, and the cell cycle. Results showed that pinocembrin inhibited cell growth and decreased colony formation of PC-3 cells in a dose-dependent way. It also regulated the expression of caspase-3, caspase-9, Bax, and Bcl-2, thus promoting apoptotic cell death in PC-3 cells. Additionally, it caused dose-dependent G0/G1 cell cycle arrest [150].

### 4.3. Flavones

Among the wide variety of flavonoid molecules, significant findings have been achieved by studying the roles of the flavones luteolin and luteolin-7-O-glucoside (Lut-7-G). Both luteolin and Lut-7-G have been shown to downregulate IL-1β, IL-6, and TNF-α, directly inhibiting JAK/STAT and other inflammatory pathways by reducing inflammation in a cellular model [151]. Acacetin, a flavone found in various plants, may directly inhibit STAT3 activation in STAT3-activated DU145 PC cells [152].

At various concentrations, acacetin inhibits DU145 cell proliferation and growth by decreasing STAT3 phosphorylation. This results in apoptosis by reducing the expression of STAT3 target proteins, including Bcl-2, Bcl-xL, Mcl-1, cyclin D1, and survivin. Acacetin also demonstrates anticancer activity by directly binding to the SH-2 domain, a region found in many signaling proteins, including Src tyrosine kinase and STAT3 [147]. Another important flavone, genistein, shows promising results in cell line studies, including PC-3 and DU145 cells. Pretreatment with genistein and AG1024 can significantly enhance inhibition of cell proliferation and promote radiotherapy-induced apoptosis. This effect is mediated by upregulating Bax and cleaved caspase-3, leading to reduced colony formation [153].

**Figure 2:**
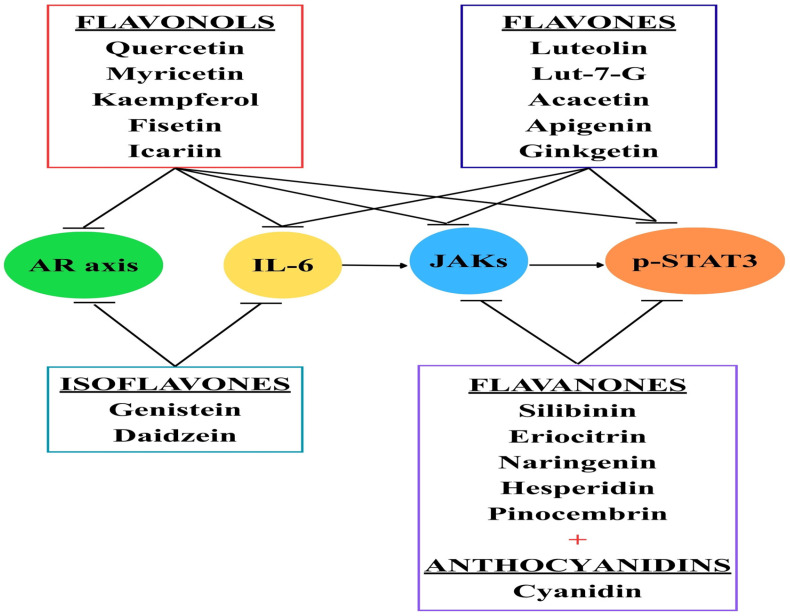
Flavonoids and their effects. Currently, different types of flavonoids are being tested, especially on PC cell lines, with promising results. Flavonols can block JAK receptors, phosphorylated STAT3, IL-6, and even the androgen receptor (AR) axis. Flavones, flavanones, and anthocyanins can inhibit the JAK/STAT3 pathway. Isoflavones target the AR axis and inhibit IL-6.

Apigenin, another important flavone, has been shown to have broad anticancer effects across various cancer types, including colorectal, breast, liver, lung, melanoma, prostate, and osteosarcoma. In PC treatment, exposing the androgen-refractory human prostate cancer cell lines PC-3 and DU145 to apigenin induced apoptosis and decreased cell viability by lowering Bcl-2 and Bcl-xL levels, while increasing the active form of Bax protein [154]. Moreover, besides apoptosis, apigenin exhibits its antitumoral effects through inhibition of angiogenesis, tumor suppressor genes, cell cycle, NF-κB, and JAK/STAT3 [155]. Promising results have been obtained with ginkgetin, a natural non-toxic biflavone [156], which inhibits both inducible and constitutively active STAT3 and blocks the nuclear translocation of p-STAT3 in DU-145 PC cells. Additionally, ginkgetin induces dephosphorylation of STAT3 at Tyr705 and prevents its nuclear localization, leading to the suppression of STAT3 target gene expression, including cell-survival-related genes (cyclin D1 and survivin) and anti-apoptotic proteins (Bcl-2 and Bcl-xL) [157] (Figure 2).

### 4.4. Isoflavones

Stanisławska et al. studied the combined effects of isoflavonoids (genistein, daidzein) and minerals (zinc, selenium, copper, iron, and calcium) on the growth of LNCaP PC cells. The results showed that flavonoids and minerals inhibited PC cell growth [158]. Some phytoestrogens, such as daidzein, genistein, and glycitein, were linked to a reduced risk of PC [159].

Davis et al. examined how genistein affects cell growth and PSA levels in a PC cell line, VeCaP, which produces PSA independently of androgens. The study showed that genistein inhibits cell growth in VeCaP cells but has different effects on PSA expression. Specifically, only high doses of genistein reduced PSA levels in VeCaP cells [160]. In PC cells, daidzein and genistein showed a synergistic effect, inhibiting cell proliferation and inducing apoptosis in both early-stage androgen-dependent cells (LNCaP) and bone-metastatic LNCaP-derived cells [161]. Daidzein can reduce the expression of inflammatory factors, including IL-1β, IL-6, and TNF-α, in a dose-dependent manner in endotoxin-induced acute liver injury [162].

### 4.5. Anthocyanidins

Anthocyanidins and anthocyanins are plant pigments mostly found in berries, pomegranates, grapes, and dark-colored fruits, which can suppress tumor cell development [163]. An important anthocyanidin from nature [164], catechin extracted from green tea, inhibits PC progression and reduces NF-κB activity [165]. Moreover, Bettuzzi S confirmed that administering green tea catechins to mice reduced oxidative incidence at 24 weeks from 100% to 20% without any side effects [166]. Cyanidin-3-O-β-glucopyranoside (C3G) has anti-proliferative activity and promotes cytodifferentiation. Treatment of DU145 and LnCap human PC cell lines with C3G reduces the number of viable PC cells after 48 h of exposure compared to vehicle-treated cells [167]. In nucleus pulposus cells, Bai et al. illustrated that cyanidin treatment downregulated the expression of p-JAK2 and p-STAT3 [168]. Therefore, it is possible that, even in PC, cyanidin also exerts similar effects and reduces levels of p-JAK2 and p-STAT3 [168].

### 4.6. Flavonoid Extracts

Additionally, flavonoid extracts revealed promising results. Wang H et al. studied the pharmacodynamics and mechanisms of the total flavonoid extract from *Hosta plantaginea* flower against BPH in a rat model. The study reported reduced prostate size and prostatic index, improved pathological damage to the prostate, elevated testosterone levels, decreased levels of IL-1β and IL-6, and inhibition of phosphorylated molecules such as JAK1 and STAT3 in a dose-dependent manner [117].

Feng R and colleagues tested the total flavonoids of *Hedyotis diffusa* Willd (TFHDW) for their effects in vitro on mouse PC cells RM1 and human PC cells, LNCaP, as well as in vivo using a xenograft tumor model involving the injection of RM1 cells. After TFHDW treatment, RM1 and LNCaP cells exhibited increased protein expression of the protein inhibitor of activated STAT and decreased STAT3 activity, accompanied by reduced proliferation, migration, and invasion [169].

*Chrysanthemum indicum* L. (*C. indicum*) flavonoid extract has been shown to possess anti-inflammatory and anticancer properties. It demonstrated strong cytotoxic activity compared to other fractions and clearly suppressed constitutive STAT3 activation in two human prostate cancer cell lines, DU145 and U266 [170]. Furthermore, the suppression of constitutive STAT3 activation by the methylene chloride fraction of *C. indicum* can inhibit JAK1 and JAK2 [170].

In conclusion, flavonoids are a key group of bioactive compounds present in plant-based foods and drinks, recognized for their effects in cells [171]. Approximately 8000 molecules from the flavonoid class are known to date and are potential candidates for discovering anticancer drugs [172]. Unfortunately, these compounds come with certain limitations, such as low absorption, decreased potency, and some side effects, despite their significant benefits [173]. Acidic conditions, light, heat, and oxidation can break down flavonoids, decreasing their potency and shelf life [174].

In recent years, nanoparticles (NPs) have become a promising method to improve the delivery and targeting of flavonoids. Encapsulating flavonoids within NPs not only protects them from degradation but also increases their solubility, leading to better absorption in the gastrointestinal tract. Additionally, the size, shape, and structure of NPs can be precisely adjusted to enhance polyphenol delivery and biocompatibility [175].

## 5. Conclusions

Prostate cancer (PC) remains a major global health concern, with higher rates seen in countries like Africa, Latin America, and the Caribbean, where men often have a genetic predisposition. In addition to genetic factors, family history, race, and ethnicity, various environmental factors also significantly influence PC development. Conditions such as obesity, smoking, sexually transmitted infections, and diet can cause chronic inflammation. This inflammation promotes the release of pro-inflammatory molecules like IL-6, which, along with growth factors and androgen imbalances, leads to overactivation of the JAK/STAT3 pathway. Dysregulation of JAK/STAT3 impacts the prostate and encourages malignant transformation. Some inhibitors targeting IL-6 or the JAK/STAT3 pathway have been tested and show promising results, including slowing or stopping cancer progression. Currently, flavonoids can inhibit molecules such as the AR axis, IL-6, JAK1, JAK2, TYK2, or STAT3 in a dose-dependent manner, demonstrating promising effects—especially in PC cell lines. Therefore, consuming flavonoids may offer health benefits related to the prostate gland.

## Data Availability

No new data were created or analyzed in this study. Data sharing is not applicable to this article.

## Abbreviations

The following abbreviations are used in this manuscript:

PC	Prostate cancer
JAK	Janus Kinase
STAT	Signal transducer and activator of transcription
TYK2	Tyrosine kinase 2
EGFR	Epidermal growth factor receptor
HGFR	Hepatocyte growth factor receptor
PDGFR	Platelet-derived growth factor receptor
FPC	Familial prostate cancer
AR	Androgen receptor
LH	Luteinizing hormone
SHBG	Sex hormone-binding globulin
DHT	5α-Dihydrotestosterone
ARE	Androgen response element
BPH	Benign prostatic hyperplasia
PIN	Prostatic intraepithelial neoplasia
NEPC	Neuroendocrine prostate cancer
mCRPC	Metastatic castration-resistant prostate cancer
Fatal PC	Fatal prostate cancer
MCs	Mast cells
NK	Natural killer
TME	Tumor microenvironment
IFN-γ	Interferon-γ
gp130	Glycoprotein 130
MAPK	Mitogen-activated protein kinase
PI3K	Phosphatidylinositol 3-kinase
IL-6R	IL-6 receptor
VEGF	Vascular endothelial growth factor
EMT	Epithelial/mesenchymal transition
LPS	Lipopolysaccharide
PD-L1	Programmed death-ligand 1
TILs	Tumor-infiltrating lymphocytes
SOCS	Suppressor of cytokine signaling
lncRNAs	Long noncoding RNAs
BNL	Borneol
DNMT	DNA methyltransferase
HAT	Histone acetyltransferase
HDAC	Histone deacetylase
ICA	Icariin
Lut-7-G	Luteolin-7-O-glucosidase
C3G	Cyaniidn-3-O-glucopyranoside
TFHDW	Total flavonoid *Hedyotis diffusa* wild
*C. indicum* L.	*Chrysanthemum indicum* L.
NPs	Nanoparticles
